# Supplementary material for: Drivers of disengagement from care during the first six months on antiretroviral therapy for HIV in South Africa: A mixed-methods study
Source: PLOS Glob Public Health. 2025 Dec 23;5(12):e0004977. doi: 10.1371/journal.pgph.0004977 (PMC12725671; doi:10.1371/journal.pgph.0004977)
Supplement: Attachment — Submitted filename: Responses PGPH.pdf [file pgph.0004977.s007.pdf]

Health Economics and Epidemiology Research Office

Unit 2, 39 Empire Road
Parktown, Johannesburg, 2193, South Africa
Tel +27 (0)10 0017930

A division of the Wits Health Consortium, a wholly own subsidiary of the University of the Witwatersrand.

27 October 2025
Editor-in-Chief: Dr. Catherine Kyobutungi
PLOS Global Public Health

Dear Dr. Kyobutungi and members of the Editorial Board,

Thank you for the opportunity to revise and resubmit this manuscript. We have addressed all the journal requirements and detail responses below. I will continue to serve as corresponding author on this submission.

1. Please provide a detailed online Financial Disclosure statement. This is published with the article. It must therefore be completed in full sentences and contain the exact wording you wish to be published.
a) State the initials, alongside each funding source, of each author to receive each grant, if applicable. For example: "This work was supported by the National Institutes of Health (####### to AM; ###### to CJ) and the National Science Foundation (###### to AM)."
b) State what role the funders took in the study. If the funders had no role in your study, please state: "The funders had no role in study design, data collection and analysis, decision to publish, or preparation of the manuscript."
For more information, please see our guidelines:
https://journals.plos.org/globalpublichealth/s/submission-guidelines#loc-financial-disclosure-statement
Response: The financial disclosure statement has been updated as requested.

2. Please ensure that the funders and grant numbers match between the Financial Disclosure field and the Funding Information tab in your submission form. Note that the funders must be provided in the same order in both places as well.
Response: The funding information statement in the manuscript text now matches the online submission tab text.

3. Please provide separate main figure files in .tif or .eps format only and ensure that all files are under our size limit of 10MB. For more information about how to convert your figure files please see our guidelines: https://journals.plos.org/globalpublichealth/s/figures
Response: Figure 1 has been submitted as .tif file

4. We have noticed that you have uploaded Supporting Information files, but you have not included a list of legends. Please add a full list of legends for your Supporting Information files before or after the references list.
Response: Legend list for supporting files is now included in the revised submitted manuscript

Health Economics and Epidemiology Research Office

Unit 2, 39 Empire Road
Parktown, Johannesburg, 2193, South Africa
Tel +27 (0)10 0017930

A division of the Wits Health Consortium, a wholly own subsidiary of the University of the Witwatersrand.

I trust these revisions will address the queries raised. I will continue to serve as corresponding author on this manuscript.

Sincerely,

Mhairi Maskew, MBBCh, PhD
Principal Researcher, Health Economics and Epidemiology Research Office
University of the Witwatersrand
