# Supplementary material for: Drivers of disengagement from care during the first six months on antiretroviral therapy for HIV in South Africa: A mixed-methods study
Source: PLOS Glob Public Health. 2025 Dec 23;5(12):e0004977. doi: 10.1371/journal.pgph.0004977 (PMC12725671; doi:10.1371/journal.pgph.0004977)
Supplement: Attachment — Submitted filename: Reviewer responses 2025 10 06.pdf [file pgph.0004977.s008.pdf]

Comment	Response
**Journal requirements**	
1. Please send a completed 'Competing Interests' statement, including any COIs declared by your co-authors. If you have no competing interests to declare, please state "The authors have declared that no competing interests exist". Otherwise please declare all competing interests beginning with the statement "I have read the journal's policy and the authors of this manuscript have the following competing interests:"	The Competing Interests statement has been updated with the requested text.
2. We noticed that you used "data not shown" in the manuscript. We do not allow these references, as the PLOS data access policy requires that all data be either published with the manuscript or made available in a publicly accessible database. Please amend the supplementary material to include the referenced data or remove the references.	We cannot find an instance of "data not shown" in our manuscript or supplementary tables.
3. Thank you for uploading your study's underlying data set. Unfortunately, the repository you have noted in your Data Availability statement does not qualify as an acceptable data repository according to PLOS's standards.<br>At this time, please upload the minimal data set necessary to replicate your study's findings to a stable, public repository (such as figshare or Dryad) and provide us with the relevant URLs, DOIs, or accession numbers that may be used to access these data. For a list of recommended repositories and additional information on PLOS standards for data deposition, please see https://journals.plos.org/plosone/s/recommended-repositories.	We have amended the repository to be used to Open Science Forum (one of the PLOS recommended platforms - https://osf.io/). As noted in our previous submission, data generated by the study will be made publicly available in this repository after the PREFER study protocol has been closed (anticipated closure December 2026). Until then, data will remain under the supervision of the Boston University Medical Campus IRB and the University of the Witwatersrand Human Research Ethics Committee (HREC). Requests can be sent to the BUMC IRB at medirb@bu.edu.
4. If the reviewer comments include a recommendation to cite specific previously published works, please review and evaluate these publications to determine whether they are relevant and should be cited. There is no requirement to cite these works unless the editor has indicated otherwise.	Thank you; this is noted.

5. Please review your reference list to ensure that it is complete and correct. If you have cited papers that have been retracted, please include the rationale for doing so in the manuscript text, or remove these references and replace them with relevant current references. Any changes to the reference list should be mentioned in the rebuttal letter that accompanies your revised manuscript. If you need to cite a retracted article, indicate the article's retracted status in the References list and also include a citation and full reference for the retraction notice.	Thank you; the reference list has been checked for completeness and accuracy. We've provided an updated link for reference 36 that is functional. Reference 38 was found to be a duplicate of reference 8 and we've changed the in-text citation to address this and remove the duplicate reference.
**Reviewer 1**	
Thank you for the opportunity to review this manuscript on the very important topic of early engagement on ART. The paper is very well written and mostly clear but I have some minor clarifying comments and edits.	Thank you for the encouraging remarks.
Abstract	
The preference data is mentioned in the methods but not the results. Could add to the "no differences" sentences for completeness.	We have added to the abstract that there were no differences by preferences for HIV care.
Intro	
Para starting line 42 – The way the groups are currently written it is not immediately clear if these are considered as mutually exclusive groups or more general risk groups that people may fall into. Someone may be a new or restarter with low CD4 for instance.	Thank you for pointing this out. We have now revised the description of these categories to clarify that those in the first group are treatment-naïve clients with low CD4 counts, the second group are treatment naïve initiators with higher CD4 counts, and the third group are treatment-experienced clients with varying of CD4 counts
Also in this paragraph – perhaps add something to make clear why these groups are important. You have already spoken about how past interruption makes people more at risk for future interruption but what about the CD4 piece. Is starting ART at a low/high CD4 will be associated with higher risk of subsequent disengagement?	Thank you. We have amended the paragraph immediately following this one to clarify the reason these groups are important is due to their high risk of disengagement and the fact that none of the service delivery innovations implemented to date address the challenges of the early treatment period.
Methods	
Study site – could you specify which provinces were included? With the large number of clinics I wondered if there were any differences by geography in any way. I see there were no differences in engagement by urban rural. Were there differences in the qualitative findings by urban/rural?	We have added text clarifying the three provinces included were Gauteng, KwaZulu-Natal and Mpumalanga. Qualitative responses did not differ substantively by urban and rural sites. We have clarified this in the methods on page 12 (first paragraph).

ART start groups - initiating, re-initiating, or on ART for ≤6 months: can you provide a little more information on the distinction between the on ART <=6 months groups and initiating group. Was there a minimum cutoff, perhaps after their first ART refill? Were any of these people very close to their ART start date (perhaps coming back for a blood result after 2 weeks or in clinic for something else shortly after). I understand this group is different potentially as you describe in the paper but it would help to describe the range of time they were on ART at enrolment.	We enrolled clients any time in the first six months on ART, as indicated in the text. There was no minimum cutoff within the 6-month period; interviews could occur at any visit during that period, though our aim was to enroll as close to initiation as possible. Unfortunately, data distinguishing between naïve initiators and re-initiators; while we did ask this question, few participants chose to disclose prior exposure to ART, and medical records do not indicate it. The range of time on ART at study enrollment is now broken in down in Table 2 (Demographics) between 0-3 months and 4-6 months.
Line 135 – counted tfo as continuously engaged but we know that many people get lost in transfer process. What about silent transfers – how common are they in your study settings?	Yes, attrition during the transfer process does occur. Overall, only 4% of the study population were reported in their medical records to have transferred out. It is difficult to accurately quantify silent transfers using facility-based data sources. Other studies using lab data have indicated that silent transfers may account for up to 25% of those considered disengaged in care over the first 5 years on treatment but this is not well quantified for the first 6 months in care. To the extent that silent transfers such as these have occurred during the first 6 months, our results will over-estimate rates of disengagement. This is noted in the limitations section of the discussion.
Results	
Line 249 – should this be among those who had a viral load (n=X)?	Thank you; the text has been corrected accordingly.
Similarly in line 259-261 it would be helpful to specify the number of people who had viral loads available. And in line 285 where you speak about predictors of VL<50.	Thank you. We have added a sentence in line 271 to clearly state the number of participants who had a VL test result: "*Out of the 1,049 participants, 705 (67%) had a VL test result available"*<br>We have also revised the numbers in the text describing % VL suppression (page 16 second last paragraph) to use those who had VL test result as the denominator, rather than the overall sample.<br>In addition, under the "predictors of viral load suppression" section (page 19 last paragraph) we have specified that results refer to those who had a VL test result available.
Disengagement was defined as 28 days late for a scheduled visit. How accurate are the Tier.net data on scheduled visits and/or quantities of drug dispensed? I know the duration of follow-up is quite short but did results differ at all if you used a slightly longer window?	We used observed visit dates from Tier.net to estimate the disengagement outcome as we expect an observed visit to have high accuracy that the visit did indeed occur on the date reported, particularly during the first 6 months when clients are not eligible for ex-facility medication collection points. However, as we note in the discussion's limitation section, not observing a visit does not necessarily indicate disengagement due to silent transfers. We selected the 28-

	day window to align with PEPFAR reporting windows but the width of the window for considering a visit attended does not impact the likelihood of outcome misclassification die to a visit not being recorded at all.
Did transfers differ at all by the predictors such as gender? I wonder if mobility, particularly unplanned, may be more common among in some groups, perhaps among men? Not to say that these people have not disengaged but there might be misclassification in assuming the transfers are retained and assuming the disengaged have not silently transferred. As raised earlier, it would be worth speaking to this in the discussion.	As mentioned above, we can only report on the handful of participants who transferred formally (not silently), as only formal transfers are captured in medical records. We did note that the transfer out group were 73% female; very similar to the overall population (71% female). We agree that misclassification is still possible and have noted this in the limitations section.
In the predictors section please add the RR and 95%CI for the age and cd4 associations that you mention in the text, even though they are not statistically significant, for consistency.	These estimates have been added to the text.
There is also some inconsistency in reporting the predictors of viral suppression, sometimes reporting % and sometimes RR and sometimes not reporting the results (CD4). This should be made consistent for ease of reading.	Thank you for pointing this out; we have revised the text to include the % as well as RR for each result reported.
Did you compare the demographic characteristics of people with and without viral loads? Any differences in these groups? It would be good to see this in a supplemental table.	We have added a stratification of participant characteristics by VL test result availability to the supplementary materials (supplementary table 5) as well as the Retention and viral load suppression outcomes section (page 15 last paragraph.
Line 358 typo: bring should be being	Thank you for catching that typo; it has been corrected now.
Were you able to draw out any differences/similarities in the qualitative findings based on the groups (new/restart/<=6m) and predictors in the quant analyses? It would be interesting to know if the qual findings differed at all by these subgroups or whether certain challenges appeared to come up more/less. In table 5, could you include the ART start group, age or age category, and gender of the participant?	Thank you for this insightful comment and we agree that it would be really interesting to know. Unfortunately, we never intended to be able to identify individuals in the FGS. The transcripts are captured in a way that allows us to differentiate participants by their assigned “Letter”, but that was only so that we could ensure no one participant was dominating our analysis or illustrative quotes. We have confirmed with source documents that there is no way to do this.<br><br>We’ve added this to the limitations: “Lastly, because FGDs were not stratified by initiating sub group (new, restart, <=6 mo) and we are unable to link individual responses within an FGD to their demographics, we could not examine differences within ART initiation sub-groups.”

**Reviewer 2**	
Congratulations on this interesting and important study that focuses on patients' experience during the early period after starting or re-starting ART. I enjoyed reading it. I have a few comments and questions that I hope will help the authors improve an already strong manuscript. I also noted a few minor language issues that need attention. Once addressed, I believe the manuscript will be appropriate for publication and has potential to add to the evidence on why individuals so frequently fail to remain on ART after beginning or re-starting treatment.	Thank you for the helpful review and encouraging remarks.
Abstract	
1. Page 2, lines 19-20: the Results section mentions alignment of findings with the socio-ecological model, but no mention of the model is made in the Methods. Could the authors describe (even briefly) the relevance of a socio-ecological model in analyses?	Thank you. We have added this to the abstract.
Introduction	
2. Page 4: the paragraph starting with "In addition to the challenges associated with…" is not easy to follow because the 3 categories of "initiators" are distinctly different. Categories 1 and 3 are genuine initiators, but category 2 are described as individuals who are restarting following a lapse on treatment, so they are not truly "initiators." It's clear this manuscript will group these three categories for the purposes of the present analysis, but it would be a bit easier to read and follow if the description of the groups began with the true initiators (categories 1 and 3) and then explained that a third group (the re-starters) may be viewed as a type of treatment starter to better understand what people experience when they either start or re-start treatment. This flow seems more logical and would be easier to follow.	Thank you for this helpful suggestion; we have rearranged the text as suggested and revised it for clarity.
Methods	

3. Page 6, line 84: how were the 18 healthcare facilities selected, and where were they located (e.g. nationwide or in specific areas)? Their client volumes were large, but can the authors describe other features that factored into the selection process so readers can better appreciate the study design and how similar the facilities were (e.g. public vs private, separate facility vs part of a larger hospital, in a rural vs urban setting)?	We have amended the text to clarify that PREFER was conducted at 18 public-sector healthcare facilities across three provinces in South Africa (Gauteng, KwaZulu-Natal and Mpumalanga) that had large ART client volumes and utilized the national electronic medical record system for HIV (TIER.Net). Sites were selected that were geographically accessible and jointly provided diversity in setting (rural, urban), facility size, and nongovernmental support partners. We have also cited the protocol paper for PREFER, which contains additional details about site selection.
4. Page 7, lines 101-102: The sentence beginning with "We note that new guidelines were issued in 2023 and have revised the schedule for the early treatment period…." is a bit confusing – did the guidelines or the authors revise the schedule (reads like the latter but likely not accurate)?	Thank you for pointing this out; the guidelines revised the schedule. We have amended the text to clarify that new guidelines were issued in 2023 which revised the schedule for the early treatment period and eligibility criteria for less intensive models of care.
5. Page 8, lines 127-128: what was the role of the second study research assistant?	Thank you. We have clarified that the second research assistant observed and took notes.
6. Page 9, lines 164-167: "As the PREFER study aimed to understand client perceptions and experiences of HIV care throughout the first six months on ART, the study enrolled participants who had initiated ART up to 6 months prior to the study enrollment to ensure that the experiences and challenges of clients who had been accessing HIV care service delivery would be observed." This describes an important feature of the design, and the purpose of the study. It would be helpful to readers if this point was made earlier in Methods or at the end of the Introduction to help clarify the purpose of the study and the range of participants being enrolled.	This description has been moved to the methods text under the "Study design" section to clarify this enrolment characteristic and the purpose thereof. We also revised the text in the Sensitivity analysis section to focus on the implication of this enrolment strategy in terms of selection bias.
7. Pages 10-11, lines 184-194: There are many specific SEMs (e.g. McLeroy or the one developed by the CDC/NIH, to name two). Can the authors be more specific as to which one they employed for the analysis, why they chose it, and provide a citation?	Thank you. We have clarified that we used McLeroy's version of the SEM and included the citation.
Results	
8. Page 12, lines 225-226: Table 2 provides data for the final linked sample and the qualitative FGD sample. Can the authors clarify what the "analytic sample" refers to	The analytic sample refers to the 1,098 participants enrolled in the PREFER study. The final linked sample refers to the 1,049 participants for whom medical records were successfully linked to the electronic medical register used to ascertain study outcomes. This relationship is depicted

in the sentence beginning with "The final linked sample….?	in Figure 1 and we have revised the text describing the creation of these samples in the text.
9. Page 13, Table 2: seeing the data on ART status at enrolment is a bit surprising given the attention to and language earlier in the Introduction on "initiation." As it turns out, the study enrolled a far higher proportion of clients on already on treatment (57%) and re-engaging (15%) than actual ART initiators (28%). The vast majority of study participants thus had at least some experience with ART at the time of enrolment. The possibility that this would characterize the final study sample could be highlighted earlier in the description of the design and enrolment plan to alert readers that the study isn't actually focused on first-time treatment initiators but rather on individuals either in an early stage of treatment or returning to care after some unknown prior period on ART (which might have been lengthy).	Our focus in PREFER was the early treatment period (0-6 months), not treatment initiation per se. This is explained in the "study sites and population section," where it states, "The study sequentially enrolled clients who were ≥18 years old, initiating, re-initiating, or on ART for ≤6 months." We have added another sentence in the paragraph following Figure 1 to reiterate the data in Table 2.<br><br>It is correct that more than half of our participants had been on ART for more than one day at study enrollment. We apologize that the text suggested that the focus was initiators only. We refer to the period of interest as "the first 6 months" rather than initiation itself, as in the title. As noted above, we know little about prior ART experience among those returning to care and so cannot comment on the prior period referred to.
10. Page 18, lines 303-306. The sentence beginning with "Neither of these two…." is interpreting the results, which seems more appropriate for the Discussion section. Please consider moving it or elaborating on this point there.	Good catch - thank you. We agree that it would generally fit in the discussion section since it is an interpretation of the results. In this case, however, triangulating with the demographic data provides the necessary context for the results within that section. We have reworded it slightly and directed the reader toward the Table 2 results.
11. Page 19, Table 5. There are numerous typos and grammar errors in the direct statements in the table. (Example: the second direct statement starting with "The medication intake…." where there are commas in places that should have periods. Another example: "My file was missing, I always have a new file and on subsequent visits the files will be all together and you just wonder where came from now." This doesn't make sense as written.) Given that the statements are the result of translations, it would be respectful to participants to present the statements here with correct grammar.	Thank you. We agree. We have edited the direct statements lightly for grammar and noted so in the methods.
Minor comments – language and grammar	
1. Page 4, line 40: The last sentence in the first paragraph of the Introduction ends with "….compared to those who had not interrupted." This wording is a bit awkward.	Thank you; we have revised the text as suggested.

Consider "… compared to those who had not experienced a treatment interruption."	
2. Page 6: The sentence beginning with "During the study period, clients presenting for ART initiation or re-initiation or on ART for less than 6 months…." has a word or two missing before "on ART".	Thank you; we have revised the text in this section.
3. Page 18, line 288. There is a missing period (".") at the end of the sentence before the one beginning with "Younger clients…."	Thank you; we have corrected this omission
